# Assessment of language abilities and functionality in adults and elders with Down syndrome

**DOI:** 10.1590/1980-5764-DN-2024-0224

**Published:** 2025-08-04

**Authors:** Cláudia Lopes Carvalho, Orestes Vicente Forlenza, Marcia Radanovic

**Affiliations:** 1 Universidade de São Paulo, Faculdade de Medicina, Hospital das Clínicas, Departamento e Instituto de Psiquiatria, Laboratório de Neurociências LIM27, São Paulo SP, Brazil.

**Keywords:** Down Syndrome, Aging, Cognition, Language, Functional Status, Síndrome de Down, Envelhecimento, Cognição, Idioma, Estado Funcional

## Abstract

Aging in people with Down syndrome (DS) is often marked by functional decline. People with DS present different degrees of language impairment, which may impact functionality.

**Objective:**

To assess language abilities and functionality in older people with DS.

**Methods:**

Forty individuals with DS aged 35 years or older were subdivided into literate and non-literate groups. The Arizona Battery for Communication Disorders in Dementia (ABCD), Object and Action Naming Battery (OANB), and Lawton & Brody Activities of Daily Living (ADL) Scale were used for linguistic and functional assessments.

**Results:**

The literate group performed better functionally and in all ABCD subdomains (apart from Linguistic Expression) and OANB. There was an association between Word Learning, Repetition, Generative Naming, and functionality in the literate group. In the non-literate group, Word Learning, Object Description, Comparative Questions, and Repetition were associated with total functional scores.

**Conclusion:**

Functional performance was associated with different linguistic tasks in the literate (verbal memory and executive functions) and non-literate (verbal memory and lexico-semantic process) groups.

## RESUMO

O envelhecimento das pessoas com síndrome de Down (SD) é frequentemente marcado por declínio funcional. As pessoas com SD apresentam diferentes graus de alteração da linguagem, o que pode afetar sua funcionalidade.

**Objetivo:**

Avaliar as capacidades linguísticas e a funcionalidade em pessoas idosas com SD.

**Métodos:**

Quarenta indivíduos com SD com idade igual ou superior a 35 anos foram subdivididos em grupos alfabetizados e não alfabetizados. As escalas Arizona Battery for Communication Disorders in Dementia (ABCD), Bateria de Nomeação de Objetos e Verbos (BNOV) e Lawton & Brody Activities of Daily Living (ADL) foram utilizadas para avaliação linguística e funcional.

**Resultados:**

O grupo alfabetizado apresentou melhor desempenho funcional e em todos os subdomínios do ABCD (com exceção da Expressão Linguística) e da BNOV. Houve associação entre Aprendizagem de Palavras, Repetição, Nomeação Generativa e funcionalidade no grupo alfabetizado. No grupo dos não alfabetizados, Aprendizagem de Palavras, Descrição de Objetos, Questões Comparativas e Repetição associaram-se à pontuação funcional total.

**Conclusão:**

O desempenho funcional foi associado a diferentes tarefas linguístico-cognitivas nos grupos alfabetizado (memória verbal e funções executivas) e não alfabetizado (memória verbal e processo léxico-semântico).

## INTRODUCTION

Down syndrome (DS), or trisomy 21, is a genetic condition resulting from the presence of all or part of a third copy of chromosome 21 in the cell nuclei that affects neurodevelopment and represents the second leading cause of intellectual disability (ID) in the world^1,2^.

Despite the numerous challenges faced by individuals with Down syndrome due to various clinical conditions that require ongoing medical attention^3,4^, this population has experienced an unprecedented increase in longevity. As a result, there is a need for adjustments in the healthcare resources provided for these individuals as they age^5-7^.

Research in cognition, language, communication, and speech has shown that individuals with DS experience altered linguistic and cognitive processing from childhood^8^ . While speech therapy can help, these difficulties may persist into adulthood and old age, particularly with the onset of dementia^9,10^.

Lack of access to proper healthcare services and limited opportunities for individuals with DS to participate actively in society can hinder the development of their skills. This impairs their functionality, makes them more susceptible to developing various diseases, and contributes to premature aging. It is estimated that 2.3% of adults with DS who are 45 years or older require total assistance from a caregiver for daily living activities; the figures are 11.9% for severe dependence, 27.9% for moderate dependence, and 8.1% for mild dependence. Cognitive decline is typically expected in late adulthood and is commonly associated with an early onset of dementia between 30 and 40 years of age^11^ . Dementia can manifest as behavioral and executive dysfunction^12^ , which usually precedes memory impairment. Cognitive decline is particularly pronounced among people with DS aged 40–49 years, with a prevalence of up to 55%. This prevalence rises to 77% in those aged 60–69 years and up to 100% in those who outlive that age^13^ .

After the age of 40, people with DS may already carry the neuropathological signature of Alzheimer’s disease (AD). Due to alterations in the metabolism of the amyloid precursor protein (APP), encoded in chromosome 21, excessive production and accumulation of beta-amyloid peptide (Aβ) occurs in the brain, triggering the pathogenic process of dementia^14^ .

In DS, assessing the relationship between cognition and functionality is particularly challenging, especially when pre-existing comorbidities like medical and psychiatric illnesses, along with sensory impairments, are present. Early signs of cognitive decline, such as deficits in executive function, working memory, and language comprehension, may impact individuals’ functional abilities performance^15^ .

The analysis of language processing in DS is complex and has not been extensively explores in the literature. It has been observed that DS can result in a distinct linguistic profile when compared to ID stemming from other causes^16^ . However, the linguistic profile in DS is highly heterogeneous^17^ .

Pre-existing language impairments in DS are classified as a specific developmental disorder (SDD)^12,18^. Individuals with DS generally exhibit better comprehension than expressive language abilities and are often competent in early vocabulary acquisition and pragmatics^19^ .

While language deficits are common in individuals with DS from early development, the aging process and the high prevalence of neurodegenerative diseases in this population can cause further declines in linguistic abilities. Over time, the progressive loss of these linguistic and communicative abilities may directly affect their functionality, reducing autonomy and social participation^9^ .

 In this study, we utilized standardized linguistic and functional assessment tools to examine the linguistic and functional performance of adults and older individuals with DS. We aimed to understand how pre-existing linguistic profiles interact with aging and relate to functionality. This knowledge may assist us in developing more effective intervention strategies to promote autonomy and enhance quality of life for this population. 

## METHODS

 This cross-sectional study was conducted at two outpatient clinics specialized in caring for people with DS, one of which was affiliated with a university hospital. The project received approval from the Research Ethics Committees at both institutions and from the National Research Ethics Committee (CONEP: 148611/2017). All research participants and/or their legal guardians provided signed informed consent. 

### Inclusion criteria

We recruited individuals who met the International Classification of Diseases 10 ^th^ Revision (ICD-10) diagnostic criteria for DS (Q.90) of both sexes who were native Brazilian Portuguese speakers aged 35 or older. To ensure that participants had the necessary cognitive abilities to perform the protocol tasks, we conducted a screening process using the Montreal Cognitive Assessment (MoCA)^20^ , the Visual Cancellation Test^21^ , and the Montreal-Toulouse Language Assessment Battery (MTL-Brazil/2016)^22^ . Participants were eligible for enrolment in the study if they could successfully complete the oral comprehension subtest of the MTL/Brazil. Individuals were deemed literate if they could perform the "Reading numbers, words, and sentences" subtest of the MTL/Brazil and write simple sentences as instructed by the examiner. We also assessed the participants’ handedness through the Edinburgh Manual Laterality Inventory^23^ .

### Exclusion criteria

- Subjective complaints or objective evidence of hearing and/or visual impairment non-correctible using prosthetic devices (for example, use of lenses);
- Evidence of recent functional decline, measured through the Informant Questionnaire on Cognitive Decline in the Elderly (IQCODE)^24,25^;
- Severe neurological and/or psychiatric impairment that impeded the performance of the proposed tasks;
- Participation in speech therapy;
- Use of supplementary alternative communication as a support for communication.

### Cognitive and language assessment

- Cambridge Examination for Mental Disorders of Older Persons with Down Syndrome and Other People with Intellectual Disabilities (CAMDEX-DS)^26,27^, which assesses general neuropsychological functioning and comprises subscales for the following domains: Orientation, Language (comprehension and expression), Memory (learning, short-term and delayed recall), Attention, Praxis (constructional, ideomotor, and ideational), Abstract Thinking, and Perception;
- An Object and Action Naming Battery (OANB)^28,29^: a visual confrontation naming task composed of black-and-white prototypical drawings of 162 objects (nouns) and one hundred actions (verbs);
- Arizona Battery for Communication Disorders in Dementia (ABCD)^30,31^, which allows the assessment of cognitive (Learning, Attention, Memory, Executive, and Visuospatial Functions) and linguistic (Comprehension and Expression) domains.

### Functionality assessment

 Lawton & Brody Scale^32^ : evaluates the level of independence in the performance of instrumental activities of daily living (IADL) through the items: Ability to use Telephone, Shopping, Food preparation, Housekeeping, Laundry, Mode of Transportation, Responsibility for own Medication, and Ability to Handle Finances. 

### Statistical analysis

 Data regarding age and performance of participants on screening, cognitive, language, and functional tests were compared between two groups (literate and non-literate) using the Mann-Whitney test, following the non-parametric distribution. The sex distribution was evaluated using Pearson’s χ ^2^ test. A hierarchical multiple linear regression was conducted to determine the best model for the association between scores on verbal tasks and functionality, examining the items in which there was a difference in performance between the literate and non-literate groups (Lawton & Brody Total score, Ability to use Telephone, Shopping, and Housekeeping) as response variables. Predictors of the model were the scores in the following ABCD tasks: Word Learning, Word Immediate Recall, Object Description, Generative Naming, Confrontation Naming, Concept Definition, Orders, Comparative Questions, and Repetition, as well as Nouns and Verbs naming from the OANB. A significance level of p<0.05 was set for all analyses. 

## RESULTS

Table 1 displays the demographic, cognitive, and functional data in the literate (n=19) and non-literate (n=21) groups. As expected, the literate group performed better in the MoCA and CAMCOG-SD and its subdomains (except for Abstraction and Perception, where no statistical difference existed between the groups). There were no differences in the perception of family members/caregivers regarding functional decline throughout life in the two groups studied, measured through the IQCODE.

**Table 1:** Demographic, cognitive, and functional data of participants by literacy group

Variable	Literate (n=19)	Non-literate (n=21)	p-value
Age	48.3 (9.1)	46.7 (8.7)	0.810
Sex			
M	12 (50%)	12 (50%)	0.698
F	7 (43.8%)	9 (56.3%)	
MoCA	20.8 (3.6)	14.9 (4.8)	<0.0001
CAMCOG-DS	75.1 (13.2)	57.0 (19.9)	0.002
Orientation	10.4 (2.5)	7.2 (3.7)	0.005
Language	20.2 (4.2)	16.5 (5.3)	0.009
Memory	16.3 (5.8)	12.2 (4.9)	0.009
Attention	7.3 (1.2)	5.0 (2.3)	0.001
Praxis	13.0 (3.9)	10.2 (3.7)	0.024
Abstraction	2.5 (2.1)	2.4 (2.1)	0.851
Perception	5.2 (1.6)	4.5 (1.8)	0.251
IQCODE	3.0 (0.6)	2.9 (0.4)	0.282
Lawton & Brody (total)	14.5 (2.7)	11.6 (2.0)	0.001
Ability to use telephone	2.7 (0.6)	2.2 (0.8)	0.044
Shopping	1.9 (0.7)	1.1 (0.3)	0.008
Food preparation	1.6 (0.8)	1.3 (0.7)	0.270
Housekeeping	2.0 (0.8)	1.4 (0.7)	0.022
Laundry	1.2 (0.4)	1.1 (0.5)	0.768
Mode of transportation	1.5 (0.8)	1.1 (0.4)	0.161
Responsibility for own medication	2.7 (0.5)	2.3 (0.7)	0.133
Ability to handle finances	1.0 (0.0)	1.0 (0.0)	1.000

Abbreviations: M, Male; F, Female; MoCA, Montreal Cognitive Assessment; CAMCOG-SD, Cambridge Cognitive Test for Down Syndrome; IQCODE, Informant Questionnaire on Cognitive Decline in the Elderly.

Notes: Data are shown as mean (standard deviation—SD), except for the variable sex.

 In the ABCD, the non-literate group performed worse in the Mental Status, Episodic Memory, and Visuospatial Abilities subdomains. Regarding language abilities, the literate group performed better in the Linguistic Comprehension subdomain, a difference linked mainly to the performance in the Repetition subtest. There was no difference between the groups in the Linguistic Expression construct, although the literate group performed better on the Naming test. Regarding the OANB scores, both nouns and verbs were named better by the literate group than the non-literate group. The literate group was better at naming nouns and verbs than the non-literate group in the OANB (Table 2). The literate group performed better on the Lawton & Brody scale, specifically in the Ability to Use Telephone, Shopping, and Housekeeping subitems (Table 1). 

**Table 2:** Performance of participants in language tests by literacy group.

Variable	Literate (n=19)	Non-literate (n=21)	p-value
ABCD			
Mental Status	7.8 (3.0)	4.5 (2.3)	0.001
Episodic Memory	52.2 (16.2)	36.8 (17.8)	0.009
Linguistic Expression Object	42.9 (13.7)	35.6 (11.6)	0.153
Description Generative	3.5 (1.9)	3.3 (1.8)	0.649
Naming Confrontation	9.2 (4.3)	6.8 (2.5)	0.124
Naming Concept	10.5 (1.9)	8.5 (2.8)	0.029
Definition	19.6 (10.7)	16.8 (9.1)	0.436
Linguistic Comprehension	57.7 (16.4)	30.5 (13.1)	<0.0001
Following commands	6.1 (1.6)	5.5 (1.5)	0.161
Comparative questions	4.1 (0.9)	3.9 (1.5)	1.000
Repetition	39.4 (14.3)	21.0 (11.5)	<0.0001
Reading comp (words)	5.2 (1.8)	NA	NA
Reading comp (sentence)	2.8 (1.7)	NA	NA
Visuospatial Abilities	11.2 (4.6)	7.0 (4.7)	0.008
OANB			
Objects	122.5 (12.8)	101.4 (28.7)	0.002
Actions	50.6 (16.8)	40.8 (15.4)	0.034
Total	173.1 (24.0)	141.9 (41.8)	0.014

Abbreviations: ABCD, Arizona Battery for Communication Disorders in Dementia; NA, not applicable; OANB, Object and Action Naming Battery.

Note: Data are shown as mean (SD).

According to the hierarchical multiple linear regression model, performance on Word Learning, Repetition, and Generative Naming tests was positively associated with Total functional performance in the literate group. Higher scores in Word Learning, Object Description, and Comparative Questions were associated with better Total Functional Performance in the non-literate group. For the literate group, the Use of Telephone subitem was associated with Word Learning, and Shopping was associated with performance in Word Learning and Repetition. In the non-literate group, the Use of Telephone subitem was best predicted by Word Learning, Object Description, and Repetition. There were no predictors for Shopping in the non-literate group or Housekeeping in any of the groups (Table 3).

**Table 3:** Hierarchical multiple linear regression results for language tests as predictors of functionality by group

Model		R^2^	R^2^ adjusted	t	p two-tailed
Literate					
Lawton & Brody (total)	Word learning	0.562	0.536	4.672	<0.0001
	Repetition	0.282	0.240	2.587	0.019
	Generative naming	0.252	0.208	2.39	0.029
Use of telephone	Word learning	0.507	0.478	4.182	0.001
Shopping	Word learning	0.323	0.283	2.846	0.011
	Repetition	0.238	0.193	2.303	0.034
Non-literate					
Lawton & Brody (total)	Word learning	0.271	0.233	2.661	0.015
	Object description	0.288	0.197	2.368	0.029
	Comparative questions	0.217	0.176	2.298	0.033
Use of telephone	39.4 (14.3)	21.0 (11.5)	<0.0001
	Object description	0.269	0.231	2.664	0.016
	Repetition	0.269	0.231	2.645	0.016

## DISCUSSION

The primary goal of this study was to investigate the association between language skills and functionality in adults and elderly individuals with DS. It is well-documented that the cognitive phenotype in DS is typically defined by neuropathological alterations in both neuronal proliferation and differentiation, which adversely affect cognitive and behavioral abilities across the lifespan^33^ . To accomplish that, we studied a cohort of adult individuals with DS, dividing them into two groups according to literacy level (literate and non-literate). Current literature on aging, cognition, and communication/language in cognitively healthy individuals highlights the importance of acquiring reading and writing abilities to develop cognitive reserve^34^ . Formal literacy and how the learning curve is shaped throughout life may be critical in successful, healthy cognitive aging.

Non-literacy in adults with DS may stem from several factors, such as learning deficits tied to the degree of prior ID, poor stimulation of metalinguistic skills, the pedagogical model implemented during the acquisition of reading and writing, life cycle and time of exposure to formal education, type and model of school attended, or simply the lack of opportunity for formal learning^35^ . Considering that oral language development (phonological awareness) is one of the prerequisites for acquiring reading and writing, it would be expected that individuals with DS may encounter greater difficulties in learning these abilities due to pre-existing deficits^36^ .

During their development, individuals with ID of various etiologies often exhibit delays in phonological and morphosyntactic processing^37^ . Challenges in the acquisition of written language are commonly observed, potentially impacting functional proficiency. Even those individuals who are literate may portray impoverished reading and writing competencies with deficits in comprehension and production of words, sentences, and texts^38^ . Throughout development into adulthood, a consistent pattern is usually maintained. Nevertheless, their capacity for language comprehension diminishes with aging, potentially disrupting daily communication–mainly when cognitive decline occurs^39, 40^.

Our results show that the literate group outperformed the non-literate in four out of five cognitive subdomains of the ABCD, except for Linguistic Expression. However, the literate group also performed better in one task within the subdomain, namely, Confrontation Naming. This result was consistent across another confrontation naming test, the OANB, where literates performed better in noun and verb naming.

Our study yielded a significant finding indicating the superior performance of the literate group in oral language comprehension and auditory perception. While the existing literature presents evidence of such deficits in children and adolescents with DS, it does not establish an association with literacy^41,42^. Linguistic Comprehension, particularly in the word-repetition task, effectively discriminated the two groups, with the non-literate subjects performing significantly worse, even though the analyses did not include reading and writing tests. Most of this disparity stems from performance on the Repetition task, where two prevalent deficits in DS are brought to the foreground: auditory perception and working memory (more specifically, the phonological loop). Thus, we can hypothesize that the differences between the two groups have their roots in fundamental oral/linguistic competencies, as well as in cognitive abilities that further support language processing.

Alterations in morphosyntactic processing may stem from deficits in working memory, particularly in the phonological loop component^13^ . Impaired oral expression in older individuals may sum up with pre-existing executive dysfunctions, leading to repercussions on phonological fluency (semantic and phonetic) and contributing to more substantial impairment in speech organization and production, resulting in difficulties in lexical and syntactic processing^43^ . Those findings underscore the need for targeted intervention early in childhood to foster the development of the two abilities mentioned above, build cognitive-linguistic reserve, and improve overall performance in language comprehension and expression.

A notable difference between the two groups was found in naming objects and verbs. A linguistic profile consisting of limited use of nouns in adults and elderly with DS is frequently found in clinical practice. Furthe more, the use of morphosyntactic markers for verbs, such as gender, number, and inflection, is often altered or effectively absent in this population^44^ .

 Functionality, as measured by the Lawton & Brody scale, was impaired for instrumental activities related to significant demands of daily life for adults and elderly with DS, with worse performance in the non-literate group. Literate individuals performed better in the tasks Ability to use telephone, Shopping, and Housekeeping. This finding highlights the importance of implementing interventions that promote autonomy and independence in individuals with developmental disabilities during childhood and early adulthood, paying particular attention to those who are not able to achieve such autonomy through formal education. Employing ecological strategies to create environments that facilitate learning is recommended^45^ . Moreover, communication and socialization are central factors in adaptive functioning and can be impacted by the linguistic deficits observed in individuals with DS. Therefore, future research could incorporate tools such as the Vineland Adaptive Behavior Scales^46^ to assess social interactions and the impact of language on global functionality more comprehensively. Including this type of evaluation may help in designing targeted interventions that improve not only functional independence but also social participation and quality of life for individuals with DS. 

 Regarding overall cognitive performance, as expected, literate DS performed better on the MoCA and CAMCOG-SD tests. However, we cannot extrapolate a cause-and-effect relationship between literacy and better functional performance based on our data (since non-literacy may also be a consequence of greater learning difficulties). Despite this, the results underscore the necessity for enhanced support and promotion of formal education for individuals with Down syndrome whenever feasible^47,48^ . 

 With respect to overall functionality and the subitems in which there were differences in performance between the two groups, our study indicated an association between Word Learning, Repetition, and Generative Naming and total scores in the Lawton & Brody scale and specific subitems (Use of Telephone and Shopping) in the literate group. In the non-literate group, the ABCD tasks Word Learning, Object Description, Comparative Questions, and Repetition were strongly associated with total scores in Lawton & Brody and the Ability to Use Telephone. 

 Word Learning emerged as the most consistent and significant ability associated with functional performance in both groups. Although Word Learning is embedded in the Episodic Memory subdomain of the ABCD, we interpreted this ability as analogous to a linguistic skill due to its assessment of short-term verbal memory. Furthermore, our findings highlight the prevalence of linguistic tasks associated with the phonological loop and executive functions (specifically Repetition and Generative Naming) as key predictors of functionality in the literate group. Interestingly, in the non-literate group (and only in this group), tasks involving lexico-semantic processing also appeared as predictors of functionality. 

 In short, our results revealed a significant association between functionality and some aspects of language impairment in adults and older persons with DS, both in terms of Language Comprehension and Expression. However, this association is conditioned by multiple factors, some of which have been discussed in this work and some that require further explorations, including a more comprehensive set of demographic variables and measures of prior cognitive performance. Our results showed that non-literate individuals performed more poorly than their literate counterparts on tests of Mental Status, Episodic Memory, Linguistic Comprehension, and Visuospatial Abilities of the ABCD, as well as on the object and actions naming task. Additionally, the non-literate group displayed worse functional performance, particularly in tasks such as using the telephone, shopping, and housekeeping. The linguistic skills most strongly associated with literate individuals’ functional abilities were those requiring auditory perception, working memory, and executive functions. At the same time, tasks demanding lexico-semantic processing were also encountered in the non-literate group. 

 The impact of illiteracy on the lifelong functioning of individuals with DS remains a complex area for analysis. However, it is undeniable that formal education and limited social engagement can impede individuals with DS from effectively achieving higher degrees of independence and autonomy in daily life. 

 One limitation of the current study was the lack of karyotyping exams, which could have enabled the genetic classification of the type of trisomy 21 in the studied group. Despite the financial costs, including these exams in research projects involving this population is advisable. Additionally, it is essential to characterize the previous degree of ID of the participants, as it can significantly influence their communication and overall functionality. Finally, it is advisable to categorize individuals into age groups (35–50 and over 50 years old), given the heterogeneous cognitive, communicative, and functional statuses of individuals with DS, which vary depending on their life stage^49^ . 

## Data Availability

The data will be available upon request.
